# Metal Phosphate-Supported Pt Catalysts for CO Oxidation

**DOI:** 10.3390/ma7128105

**Published:** 2014-12-17

**Authors:** Xiaoshuang Qian, Hongmei Qin, Tao Meng, Yi Lin, Zhen Ma

**Affiliations:** Shanghai Key Laboratory of Atmospheric Particle Pollution and Prevention (LAP^3^), Department of Environmental Science and Engineering, Fudan University, Shanghai 200433, China; E-Mails: 12210740023@fudan.edu.cn (X.Q.); 12210740024@fudan.edu.cn (H.Q.); 13110740020@fudan.edu.cn (T.M.); 13210740026@fudan.edu.cn (Y.L.)

**Keywords:** metal phosphate, hydroxyapatite, Pt catalyst, CO oxidation

## Abstract

Oxides (such as SiO_2_, TiO_2_, ZrO_2_, Al_2_O_3_, Fe_2_O_3_, CeO_2_) have often been used to prepare supported Pt catalysts for CO oxidation and other reactions, whereas metal phosphate-supported Pt catalysts for CO oxidation were rarely reported. Metal phosphates are a family of metal salts with high thermal stability and acid-base properties. Hydroxyapatite (Ca_10_(PO_4_)_6_(OH)_2_, denoted as Ca-P-O here) also has rich hydroxyls. Here we report a series of metal phosphate-supported Pt (Pt/M-P-O, M = Mg, Al, Ca, Fe, Co, Zn, La) catalysts for CO oxidation. Pt/Ca-P-O shows the highest activity. Relevant characterization was conducted using N_2_ adsorption-desorption, inductively coupled plasma (ICP) atomic emission spectroscopy, X-ray diffraction (XRD), transmission electron microscopy (TEM), CO_2_ temperature-programmed desorption (CO_2_-TPD), X-ray photoelectron spectroscopy (XPS), and H_2_ temperature-programmed reduction (H_2_-TPR). This work furnishes a new catalyst system for CO oxidation and other possible reactions.

## 1. Introduction

Heterogeneous metal catalysts are very useful in synthesizing chemicals, processing fuels, and ablating environmental pollutants [1]. Heterogeneous metal catalysts are often prepared by loading metals (e.g., Pt, Pd, Au, Ag, Rh) onto oxide or carbon supports. Typical oxide supports include SiO_2_, Al_2_O_3_, TiO_2_, ZrO_2_, Fe_2_O_3_, and CeO_2_. These supports, with good thermal stability and high surface areas (usually from 10 to 1000 m^2^/g) not only provide a platform for dispersing and stabilizing metal nanoparticles, but also render the final catalysts various properties (e.g., acid-base, redox properties). It can be said that these conventional supported metal catalysts are at the heart of heterogeneous catalysis because they are so useful in a variety of reactions and the global market for these catalysts is huge.

Compared with metal oxides and carbons, metal salts have seldom been used in the preparation of supported metal catalysts [2] because many metal salts (e.g., most nitrates, chlorides, and sulfates) are soluble in water and are not thermally stable. These attributes make the preparation of heterogeneous catalysts a daunting task. Fortunately, some metal salts (e.g., metal phosphates) are not soluble in water, and they have good thermal stability. In addition, some metal phosphates are recognized for their acid-base properties [3]. In a classic book written by Tanabe, Misono, Ono, and Hattori, metal phosphates are highlighted as a new family of solid acids showing some promising applications in heterogeneous catalysis [3]. Metal phosphates can be used as catalysts directly, for instance, in the partial oxidation of propane [4], oxidative dehydrogenation of isobutane to isobutene [5,6,7], oxidative dehydrogenation of ethylbenzene to styrene [8], dehydration of cyclohexanol and 1-methylcyclohexanol [9,10,11], retro-Prins reaction [12,13], terpene rearrangements [14], alkylation of anisole with alcohols [15], dehydrofluorination of CF_3_CH_3_ into CF_2_CH_2_ [16,17], hydrolysis of CCl_2_F_2_ [18,19,20], decomposition of CF_4_ [21,22], decomposition of CH_2_FCF_3_ [23], decomposition of chlorobenzene [24], decomposition of SF_6_ [25,26], and hydrolysis of NF_3_ [27].

Alternatively, metal phosphates can be used as supports to prepare supported metal catalysts. For instance, Lisnyak and co-workers [28] reported that the activity of several Pt and Pd catalysts in catalytic H_2_ oxidation followed the sequence of Pt(Pd)/NbP_2_O_7_ > Pt(Pd)/NbPO_5_ > Pt(Pd)/Al_2_O_3_. Johnstone and co-workers [29] investigated the hydrogenation of 1-octene, 1-,3-,4-methylcyclohexenes, and cyclododecene using Pd and Pt supported on a variety of metal(IV) phosphates. Miura and co-workers [30] demonstrated that Pd/AlPO_4_ showed high activity in the hydrogenation of naphthalene in the presence of CO. Takita and co-workers [31] employed Pt/AlPO_4_ and Pt/CePO_4_-AlPO_4_ for the hydrodechlorination of CHF_2_CClF_2_. More recently, Dai and co-workers [32,33,34] developed a series of metal phosphate-supported Au (denoted as Au/M-P-O, M = Fe, Co, Y, La, Pr, Nd, Sm, Eu, Ho, Er) catalysts and tested their performance in CO oxidation. Among them, Au/La-P-O and Au/Fe-P-O are representative catalysts, and the nature of active sites and reaction mechanisms were studied in detail [35,36]. These supported catalysts provide new opportunities for studying the nature of active sites and reaction mechanism, and for exploring their applications in other reactions.

Hydroxyapatite (HAP, also denoted as Ca-P-O here), with the formula Ca_10_(PO_4_)_6_(OH)_2_, can be used as a catalyst directly [37], for instance, in the conversion of ethanol [38,39,40], Knoevenagel condensation [41,42], aldol condensation [43], Friedel-Crafts reaction [44], Michael addition [45], and formaldehyde combustion [46]. Heteroatoms, such as Pb [47], Pd [48,49], Cu [50,51,52], Zn [53], Ni [54], V [55], Nb [56], and Sr [57] can be incorporated into the hydroxyapatite structure via ion-exchange, hydrothermal synthesis, and other methods to tune the catalytic properties.

Hydroxyapatite, with rich hydroxyls, can also be used to support metal nanoparticles. For instance, Scurrell and co-workers prepared Au/HAP for the water-gas shift reaction [58]. Several groups developed Au/HAP catalysts for CO oxidation [33,59,60,61,62]. Au/HAP catalysts were also used for the deoxygenation of amides [63], the epoxidation of styrene or cyclohexane [64], the direct tandem synthesis of imines or oximes [65], and the removal of organic compounds from aqueous medium via wet peroxidation [66]. Pd/HAP was demonstrated to be a highly efficient and recyclable catalyst for the selective reduction of carbon-carbon double bond in α,β-unsaturated ketones [67]. Rh/HAP was found to be useful for the water-gas shift reaction [58], the mild racemization of alcohols [68], *cis*-dihydroxylation and oxidative cleavage of alkenes [69], as well as hydrogen generation from ammonia-borane solution [70]. Ag/HAP was reported to have high activities for the selective oxidation of silanes into silanols using water as an oxidant [71] and selective reduction of NO_x_ by propene [72].

Supported Pt catalysts have wide applications in hydrotreating, selective hydrogenation/oxidation, conversion of biomass, ablation of environmental pollutants (e.g., CO, VOCs), and H_2_ fuel cells [1]. Although there are a number of papers on Pt-catalyzed CO oxidation [73,74,75,76,77,78,79,80], to the best of our knowledge, there was few or almost no report on metal phosphate-supported Pt catalysts for CO oxidation. There were only a few reports on the catalytic oxidation of H_2_ on Pt(Pd)/NbP_2_O_7_ and Pt(Pd)/NbPO_5_ [28], hydrodechlorination of CHF_2_CClF_2_ on Pt/AlPO_4_ and Pt/CePO_4_-AlPO_4_ [31], and electrocatalysis on Pt/AlPO_4_ [81], Pt/CePO_4_ [82], and Pt/FePO_4_ [83,84] thin film electrodes.

Following our previous work on metal phosphate-supported gold catalysts [33,34], here we developed a series of metal phosphate-supported Pt catalysts and tested their performance in CO oxidation. Interestingly, Pt/Ca-P-O shows the highest activity. Relevant characterization was conducted using XRD, N_2_ adsorption-desorption, ICP, TEM, CO_2_-TPD, XPS, and H_2_-TPR.

## 2. Results and Discussion

### 2.1. Catalytic Activity of Pt/M-P-O

**Figure 1:**
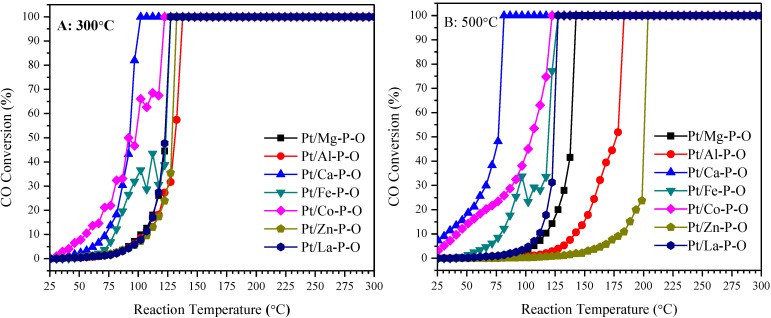
CO conversions on Pt/M-P-O catalysts calcined at 300 °C (**A**) or 500 °C (**B**) and then pretreated in 4% H_2_ at 300 °C prior to reaction testing.

Figure 1 shows the CO conversions on Pt/M-P-O (M = Mg, Al, Ca, Fe, Co, Zn, La) catalysts as a function of reaction temperature. These catalysts were prepared by calcining the impregnated H_2_PtCl_6_/M-P-O precursor at 300 or 500 °C in a muffle oven, and were pretreated in the catalytic reactor with 4% H_2_ (balance He) at 300 °C prior to reaction testing (see the Experimental section for more details). The H_2_-pretreatment, sometimes adopted in the literature [73,76,79,80] but not always [74,75,77,78], was intended to facilitate the reduction of Pt and the removal of Cl from the Cl-containing Pt precursor [85,86,87]. Our initial experiments show that the pretreatment in 4% H_2_ at 300 °C is beneficial for improving the catalytic activity in some cases, although the calcined catalysts (without further H_2_ pretreatment) are also active (Figure S1 in the Supporting Information).

As shown in Figure 1A, the CO conversion increases with the reaction temperature and reaches 100% at certain temperatures. The T_50_ (temperature required for 50% conversion) values of Pt/M-P-O (M = Mg, Al, Ca, Fe, Co, Zn, La) calcined at 300 °C and then pretreated in 4% H_2_ at 300 °C are 123, 128, 92, 123, 90, 130, and 123 °C, respectively. The temperature window for 100% conversion on these catalysts is 100–140 °C. Among these catalysts, the most active Pt/Ca-P-O achieves 100% conversion at 100 °C, whereas the least active Pt/Al-P-O achieves 100% conversion at 140 °C.

In practical applications, sometimes catalysts have to encounter high-temperature environments, e.g., during catalytic reactions or in catalyst regeneration. Therefore, to see whether the catalysts are still active after high-temperature treatment, the same batch of Pt/M-P-O catalysts were calcined at a higher temperature (500 °C) then pretreated in 4% H_2_ at 300 °C prior to reaction testing. As shown in Figure 1B, the T_50_ values of Pt/M-P-O (M = Mg, Al, Ca, Fe, Co, Zn, La) calcined at 500 °C and then pretreated in 4% H_2_ at 300 °C are 140, 178, 76, 120, 107, 202, and 123 °C, respectively. The temperature window for 100% conversion on these catalysts is 80–210 °C, indicating that they are still active after calcination at 500 °C. Note that the 500 °C-calcined Pt/Ca-P-O (T_50_ = 76 °C) is even more active than the 300 °C-calcined Pt/Ca-P-O (T_50_ = 90 °C), probably because the removal of more residual Cl at 500 °C and the enhanced metal-support interaction upon high-temperature calcination.

The difference in catalytic activity is more obvious for the catalysts calcined at 500 °C (and subsequently pretreated in 4% H_2_ at 300 °C), and the activity follows the sequence of Pt/Ca-P-O > Pt/Co-P-O > Pt/Fe-P-O > Pt/La-P-O > Pt/Mg-P-O > Pt/Al-P-O > Pt/Zn-P-O. Because the difference in catalytic activity is large for the catalysts calcined at 500 °C and large difference in activity may help us find the correlation between catalytic activity and physicochemical properties of catalysts, the following research mainly focused on these catalysts calcined at 500 °C.

### 2.2. BET Surface Areas and Pt Contents

**Figure 2:**
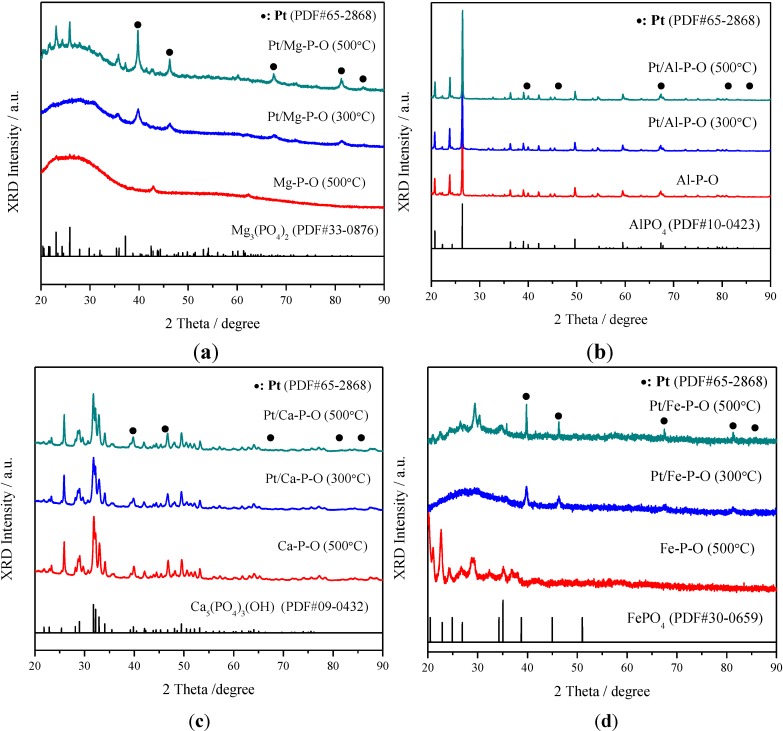

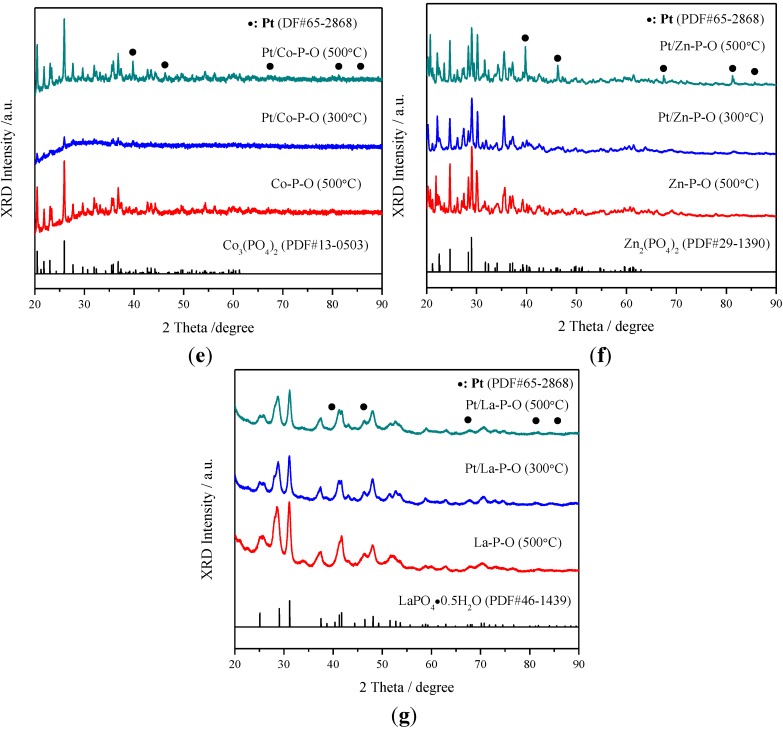
XRD patterns of spent Pt/M-P-O catalysts (calcined at 300 or 500 °C, pretreated in 4% H_2_ at 300 °C, and tested in CO oxidation) and M-P-O supports calcined at 500 °C. M = Mg (**a**), Al (**b**), Ca (**c**), Fe (**d**), Co (**e**), Zn (**f**), La (**g**).

The BET surface areas of Pt/M-P-O (M = Mg, Al, Ca, Fe, Co, Zn, La) catalysts are 24.4, 5.2, 47.1, 8.7, 7.7, <1 (below the detection limit), and 71.3 m^2^/g, respectively, *i.e*., Pt/La-P-O shows the highest surface area of 71.3 m^2^/g, Pt/Ca-P-O has the second largest surface area of 47.1 m^2^/g, whereas Pt/Zn-P-O shows the lowest surface area (<1 m^2^/g). One may question the low surface area of Pt/Zn-P-O. We previously prepared Au/Zn-P-O using Zn-P-O from another commercial supplier (Aldrich), and the surface area of Au/Zn-P-O was only 3 m^2^/g [33]. The low surface area is consistent with the bulk-like morphology of the support (see Section 2.4) and sharp XRD peaks corresponding to Zn-P-O (Figure 2).

The Pt content of these catalyst prepared by impregnation (without any washing procedure) was intended to be fixed at 2 wt%. However, some loss of Pt was found to occur during the calcination of the H_2_PtCl_6_/M-P-O precursors in porcelain crucibles. The inner walls of porcelain crucibles become shinny in some instances, indicating the coating of some metallic Pt to the inner walls. In addition, some drops of H_2_PtCl_6_ solution may adhere to the wall of a beaker during catalyst preparation. The Pt contents (wt.%) in Pt/M-P-O (M = Mg, Al, Ca, Fe, Co, Zn, La) calcined at 500 °C were determined by ICP as 0.49%, 1.35%, 0.85%, 1.42%, 1.43%, 1.23%, and 1.45%, respectively.

### 2.3. XRD Characterization

Figure 2 summarizes the XRD patterns of Pt/M-P-O catalysts (calcined at 300 or 500 °C and then pretreated in 4% H_2_ at 300 °C) collected after catalytic testing. These catalysts are referred to as spent Pt/M-P-O catalysts. Spent catalysts were characterized herein because they are closer to the working catalysts after being exposed to the reaction ambient. The XRD patterns of M-P-O calcined at 500 °C and standard M-P-O substances (with the corresponding PDF numbers) are shown for comparison.

As shown in Figure 2, Pt/Mg-P-O, Pt/Fe-P-O, Pt/Co-P-O, and Pt/Zn-P-O calcined at 500 °C (and then pretreated in 4% H_2_ at 300 °C and subjected to reaction testing) exhibit obvious Pt peaks at 2θ = 39.7°, 46.2°, 67.4°, 81.2°, and 85.6°, indicating the presence of relatively big Pt particles on these supports. The sintering of Pt nanoparticles is not so obvious for Pt/Mg-P-O, Pt/Fe-P-O, Pt/Co-P-O, and Pt/Zn-P-O calcined at 300 °C (and then pretreated in 4% H_2_ at 300 °C and subjected to reaction testing). On the other hand, spent Pt/Al-P-O, Pt/Ca-P-O, and Pt/La-P-O virtually do not show metallic Pt peaks, regardless of the calcination temperature, indicating that Pt nanoparticles are highly dispersed.

### 2.4. TEM Characterization

It is well know that the XRD technique has some limitations. For instance, when there are both small and big metal nanoparticles on a support, the XRD peak intensity is biased toward big metal nanoparticles, *i.e*., the XRD peak becomes too sharp [88]. In order to better see the microscopic picture of the catalysts, the spent Pt/M-P-O catalysts (calcined at 500 °C, pretreated in 4% H_2_ at 300 °C, and then tested in CO oxidation) were characterized by TEM. Here we classify the catalysts into two categories according to the above-mentioned XRD data that Pt/Mg-P-O, Pt/Fe-P-O, Pt/Co-P-O, and Pt/Zn-P-O calcined at 500 °C exhibit obvious Pt peaks, whereas Pt/Al-P-O, Pt/Ca-P-O, and Pt/La-P-O virtually do not show Pt peaks (Figure 2).

**Figure 3:**
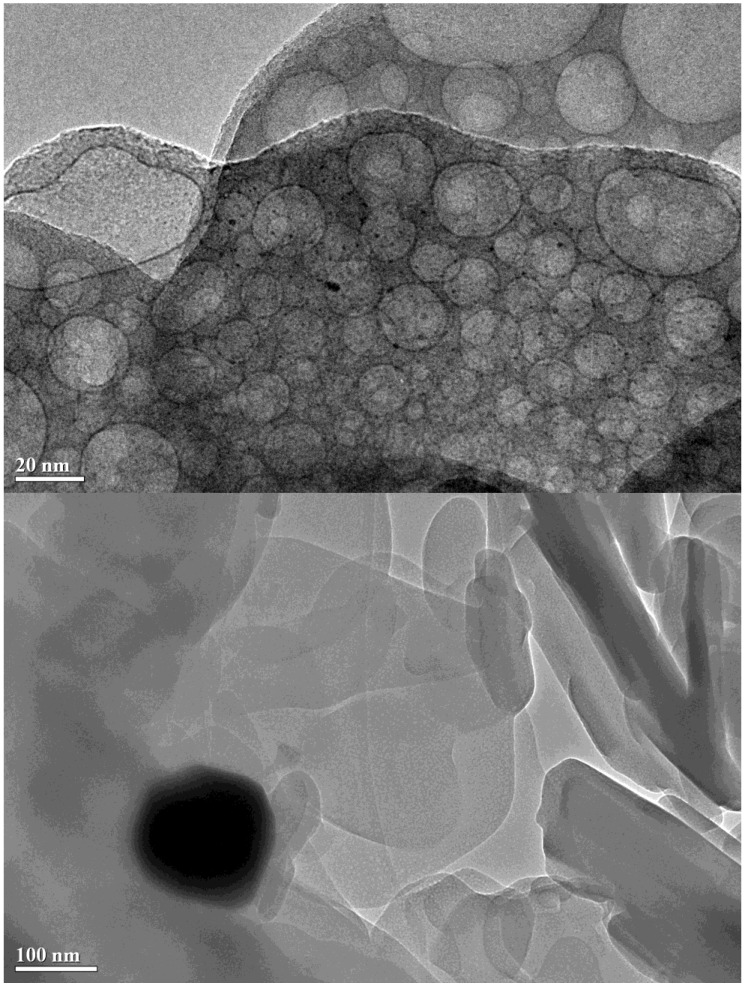
TEM image of spent Pt/Mg-P-O (calcined at 500 °C, pretreated in 4% H_2_ at 300 °C, and tested in CO oxidation).

As shown in Figure 3, Pt nanoparticles with sizes of 2–5 nm are highly dispersed on Mg-P-O (top image), whereas very big Pt particles are occasionally seen (bottom image). This observation explains the existence of sharp Pt peaks on the XRD pattern of spent Pt/Mg-P-O. Clearly, the XRD peak intensity of Pt on Mg-P-O had been biased toward big Pt nanoparticles.

As shown in Figure S2 in the Supporting Information, Pt nanoparticles (2–5 nm in size) are highly dispersed on Fe-P-O. Bigger Pt particles on the order of 10 nm are also seen. The TEM data again explain the existence of Pt peaks on the XRD pattern of spent Pt/Fe-P-O.

Similarly, Pt/Co-P-O has many small Pt nanoparticles (2–5 nm in size) and only a small portion of bigger Pt particles on the order of 10 nm (Figure S3).

On the other hand, only low-resolution TEM images of Pt/Zn-P-O were collected (Figure S4) because this sample underwent significant structural transformations under the high-energy electron beam when we attempted to record high-resolution TEM images. From the low-resolution TEM images, it is not clear whether this sample has small Pt nanoparticles or not, but big Pt particles can been seen at the edge of the support.

**Figure 4:**
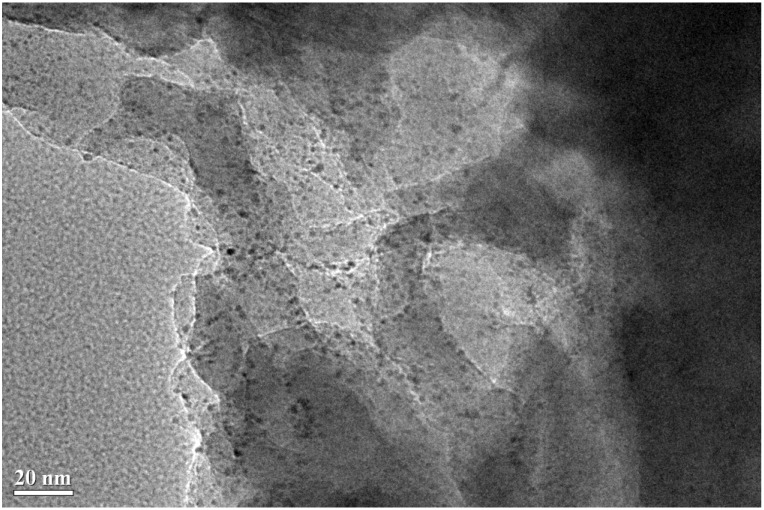
TEM image of spent Pt/Al-P-O (calcined at 500 °C, pretreated in 4% H_2_ at 300 °C, and tested in CO oxidation).

**Figure 5:**
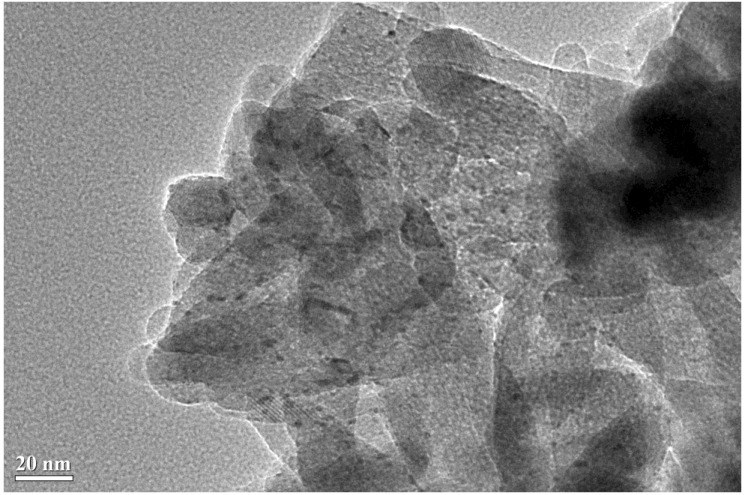
TEM image of spent Pt/Ca-P-O (calcined at 500 °C, pretreated in 4% H_2_ at 300 °C, and tested in CO oxidation).

We then turn to the TEM results of spent Pt/Al-P-O, Pt/Ca-P-O, and Pt/La-P-O that virtually do not show XRD peaks of Pt (Figure 2). As shown in Figure 4, Pt/Al-P-O has Pt nanoparticles with sizes in the range of 2–3 nm, consistent with the XRD data showing no Pt peaks. Pt/Ca-P-O and Pt/La-P-O also have small Pt nanoparticles (2–3 nm), whereas big Pt particles are virtually not seen (Figure 5 and Figure S5). Overall, the TEM data and XRD data are complementary: while XRD data show the macroscopic picture of a number of solid powders, TEM data directly show the microscopic picture of the supported Pt particles.

### 2.5. CO_2_-TPD

As shown from the catalysis data (Figure 1), Pt/Ca-P-O shows the highest activity. We hypothesize that the surface basicity of the Ca-P-O support [40] plays an important role in the high activity. Therefore, CO_2_-TPD experiments were conducted. The principle of CO_2_-TPD is that CO_2_, a weakly acidic molecule, adsorbs onto basic sites of a catalyst at a relatively low temperature (e.g., 50 °C), and then desorbs when the temperature ramps. That way, the desorption of CO_2_ can be used to characterize the basicity of a catalyst. Here, the term “basicity” consists of two parameters: the strength of base (characterized by the peak temperature of CO_2_ desorption) and the amount of basic sites (characterized by the area of the desorption peak).

**Figure 6:**
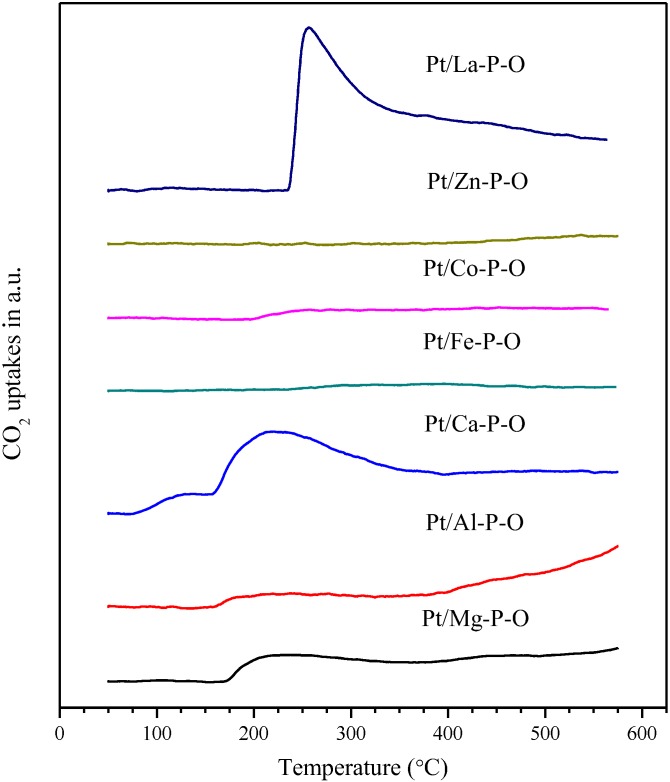
CO_2_-TPD profiles of Pt/M-P-O calcined at 500 °C and then pretreated in 4% H_2_ at 300 °C.

As shown in Figure 6, Pt/Mg-P-O, Pt/Al-P-O, Pt/Fe-P-O, Pt/Co-P-O, and Pt/Zn-P-O have very limited desorption of CO_2_, indicating the lack of basic sites on these catalysts. On the other hand, both Pt/Ca-P-O and Pt/La-P-O have obvious peaks corresponding to CO_2_ desorption. Pt/Ca-P-O has a main desorption peak centered at 204 °C, whereas Pt/La-P-O has a desorption peak centered at 257 °C. The amounts of basic sites are 76.8 and 147.9 µmol/g, respectively. That means that Pt/La-P-O not only has more basic sites than Pt/Ca-P-O, its basic strength is also stronger than that of Pt/Ca-P-O.

**Figure 7:**
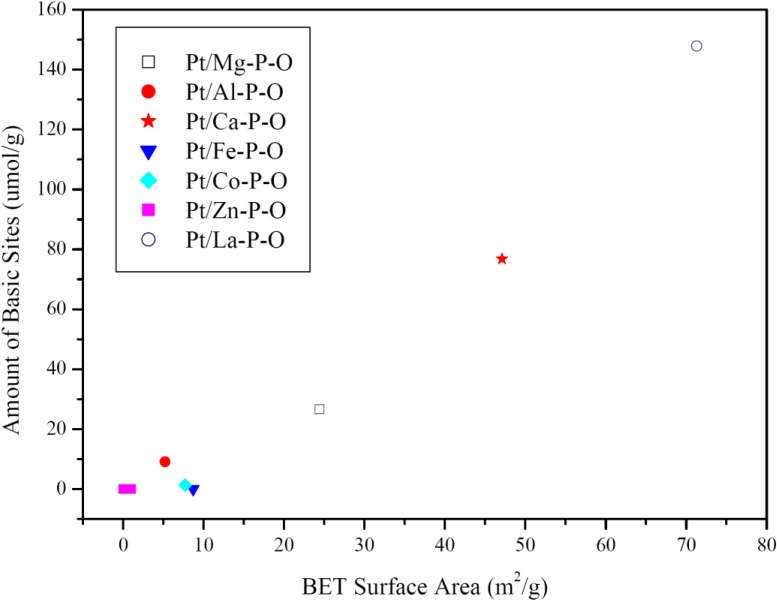
Correlation between the BET surface area and amount of basic sites of Pt/M-P-O catalysts calcined at 500 °C.

The trend seen in the amount of CO_2_ desorption is generally consistent with the BET surface area of the catalysts, being 24.4, 5.2, 47.1, 8.7, 7.7, <1 (below detection limit), and 71.3 m^2^/g for Pt/M-P-O (M = Mg, Al, Ca, Fe, Co, Zn, La) catalysts. Table 1 summarizes some catalysts and characterization results. The correlation between surface area and amount of basic sites is seen in Figure 7.

**Table 1:** T_50_ values, specific surface areas, Pt contents, average Pt particle sizes, amounts of basic sites, as well as the distribution of surface oxygen species of Pt/M-P-O catalysts. The T_50_ values refer to those of catalysts calcined at 500 °C and pretreated in 4% H_2_ at 300 °C. The surface areas and Pt contents refer to those of catalysts calcined at 500 °C. The amounts of basic sites refer to the catalysts calcined at 500 °C and pretreated in 4% H_2_ at 300 °C. The average Pt particle sizes and the distribution of surface oxygen species (O_B_: bridging oxygen; O_NB:_ non-bridging oxygen; O_OH_: hydroxyl oxygen) refer to those of catalysts collected after reaction.

Catalyst	T_50_ (°C)	S_BET_ (m^2^/g)	Pt content (%)	Average Pt particle size (nm)	Basic sites (μmol/g)	O1s (%)		
						O_B_	O_NB_	O_OH_
Pt/Mg-P-O	140	24.4	0.49	2–5, some are very big	26.6	29.8	57.0	13.2
Pt/Al-P-O	178	5.2	1.35	2–3	9.1	30.2	61.2	8.6
Pt/Ca-P-O	76	47.1	0.85	2–3	76.8	16.6	45.5	37.9
Pt/Fe-P-O	120	8.7	1.42	2–5, some are big (10 nm)	0	29.6	57.1	13.3
Pt/Co-P-O	107	7.7	1.43	2–5, some are big (10 nm)	1.3	35.1	52.1	12.8
Pt/Zn-P-O	202	<1	1.23	very big	0	20.1	66.8	13.1
Pt/La-P-O	123	71.3	1.45	2–3	147.9	32.2	59.0	8.8

Overall, the CO_2_-TPD data indicate that although an active Pt/M-P-O catalyst does not have to own basic sites, the most active Pt/Ca-P-O catalyst has plenty of weak basic sites, which may promote CO oxidation. On the other hand, although Pt/La-P-O has more basic sites, it is less active than Pt/Ca-P-O, probably because too strong basic sites may make the desorption of CO_2_ (produced by CO oxidation) difficult. As a rule of thumb in heterogeneous catalysis, too strong adsorption of the reactant/intermediate/product usually lowers the activity in catalysis [1].

### 2.6. XPS Characterization

It was reported that the presence of hydroxyls on catalyst surfaces can promote CO oxidation [89], whereas Ca-P-O has plenty of hydroxyls [40]. Therefore, we studied the distribution of oxygen species of different catalysts via XPS. The Pt/M-P-O catalysts refer to the ones collected after reaction testing. As we mentioned above, the spent catalysts collected after reaction testing are closer to the working catalysts since they were exposed to the reaction ambient. We will report the XPS data of Pt/Ca-P-O calcined at 500 °C, Pt/Ca-P-O calcined at 500 °C and pretreated in 4% H_2_ at 300 °C, as well as spent Pt/Ca-P-O collected after reaction testing later in this section.

**Figure 8:**
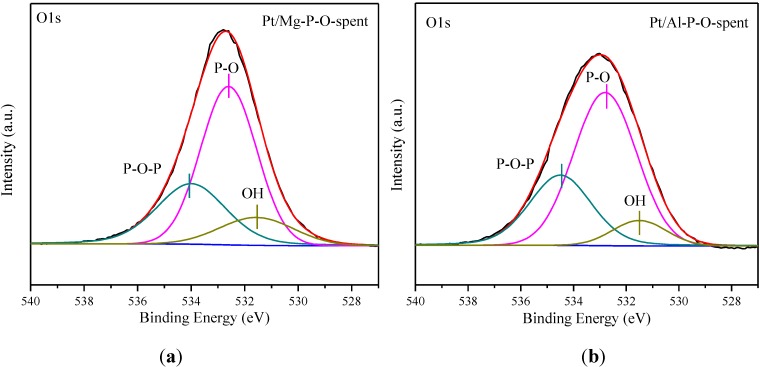

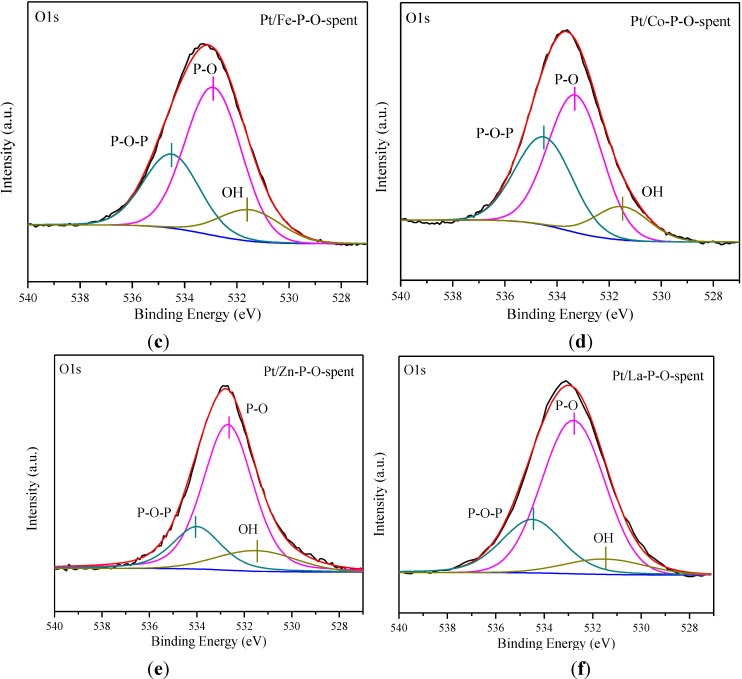
O1s XPS data of Pt/M-P-O catalysts collected after reaction testing. M = Mg (**a**), Al (**b**), Fe (**c**), Co (**d**), Zn (**e**), La (**f**).

Figure 8 shows the O1s spectra in the range of 527–540 eV. There are three kinds of oxygen species. The peak centered at 532.7 ± 0.2 eV is assigned to non-bridging oxygen (P-O), the peak centered at 534.2 ± 0.3 eV is assigned to bridging oxygen (P-O-P), whereas the peak centered at 531.5 eV is ascribed to hydroxyl oxygen (–OH) [72,90,91,92]. The original O1s spectra were then deconvoluted according to the assignments. As shown in Figure 8 and Table 1, the percentages of hydroxyl oxygen (among all surface oxygen species) in Pt/M-P-O (M = Mg, Al, Fe, Co, Zn, La) are 13.2%, 8.6%, 13.3%, 12.8%, 13.1%, and 8.8%, respectively, whereas that of Pt/Ca-P-O is 37.9% , consistent with the highest activity of Pt/Ca-P-O.

To provide more information on the nature of key elements of the most active catalyst and know more about what happens in various stages of treatment/reaction, Pt/Ca-P-O collected at several stages were characterized by XPS. “Pt/Ca-P-O-calcined” refers to the catalyst calcined at 500 °C, without further pretreatment in 4% H_2_. “Pt/Ca-P-O-pre H_2_” refers to the catalyst calcinated at 500 °C and pretreated in 4% H_2_ at 300 °C, without undergoing catalytic reaction. “Pt/Ca-P-O-spent” refers to the catalyst collected after reaction testing.

**Figure 9:**
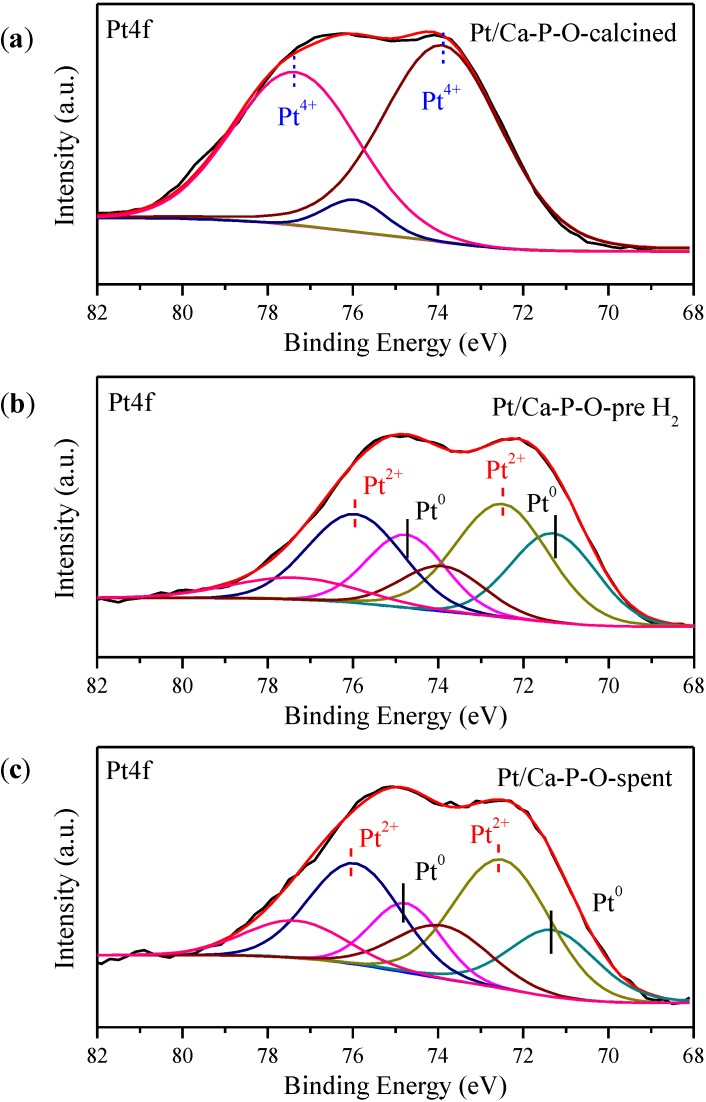
Pt4f XPS data of Pt/Ca-P-O calcined at 500 °C (**a**); Pt/Ca-P-O calcined at 500 °C and then pretreated in 4% H_2_ at 300 °C (**b**); and Pt/Ca-P-O collected after reaction (**c**).

As shown in Figure 9 and Table 2, the Pt in “Pt/Ca-P-O-calcined” mainly exists in the form of Pt^4+^, which represents 90.8% of all Pt species (Pt^0^, Pt^2+^, Pt^4+^). This is understandable, considering that the decomposition of H_2_PtCl_6_ occurs under air at 500 °C. Upon reduction in 4% H_2_ at 300 °C, the Pt species in the catalyst are mainly Pt^0^ (33.8%) and Pt^2+^ (49.5%). Certainly, the 4% H_2_ is not enough to fully reduce Pt^4+^ at 300 °C. For the catalyst collected after CO oxidation, the relative percentages of Pt^0^ and Pt^2+^ change to 27.2% and 51.7%, respectively, indicating the oxidation of a small portion of Pt^0^ during the course of the reaction. Therefore, the working catalyst contains both Pt^0^ and Pt^2+^, together with a small portion of Pt^4+^.

**Table 2:** Results from XPS analysis of Pt/Ca-P-O catalysts.

Sample	Ca/P	Pt^0^/Pt_total_	Pt^2+^/Pt_total_	Pt^4+^/Pt_total_	O_OH_ (%)	Cl/Pt
Pt/Ca-P-O-calcined	1.29	0	0.092	0.908	30.1	1.39
Pt/Ca-P-O-pre H_2_	1.29	0.338	0.495	0.167	46.1	1.26
Pt/Ca-P-O-spent	1.38	0.272	0.517	0.211	37.9	1.22

**Figure 10:**
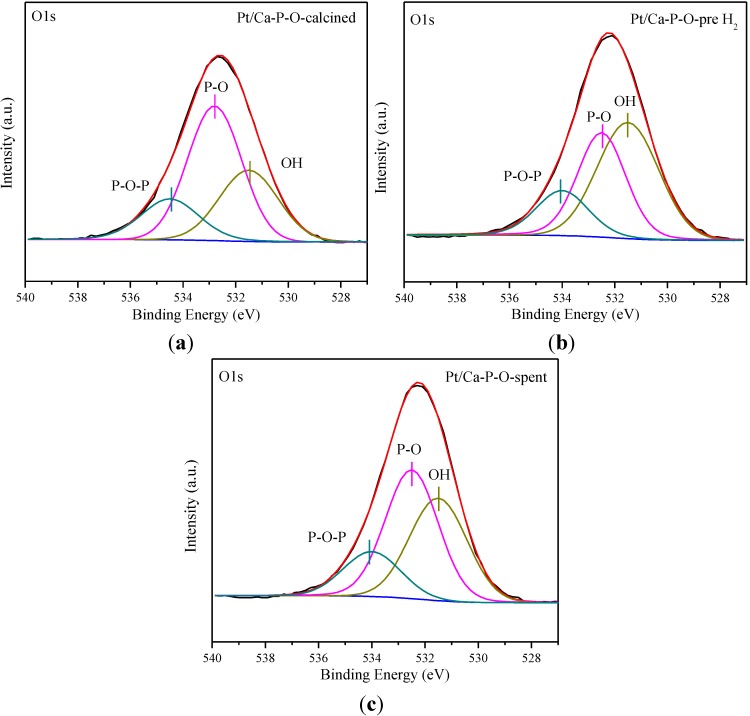
O1s XPS data of Pt/Ca-P-O-calcined (**a**), Pt/Ca-P-O-pre H_2_ (**b**), and Pt/Ca-P-O-spent (**c**).

Figure 10 shows the XPS data for O1s peaks. The broad O1s peak can be deconvoluted into three peaks centered at 531.5, 532.6, and 534.5 eV, corresponding to hydroxyl oxygen (–OH), non-bridging oxygen (P-O), and bridging oxygen (P-O-P). As shown in Figure 10 and Table 2, the percentage of hydroxyl oxygen among all oxygen species in “Pt/Ca-P-O-calcined” is 30.1%. After the catalyst was pretreated in 4% H_2_ at 300 °C, the value increases to 46.1%. This finding is interesting, in that the H_2_ treatment at 300 °C can replenish some hydroxyls. After reaction testing, the value decreases slightly to 37.9%.

Since the catalysts were prepared by impregnating H_2_PtCl_6_ onto supports, the residual Cl should be examined. According to our XPS analysis, the surface Cl/Pt ratio for “Pt/Ca-P-O-calcined” is 1.39. The number is not negligible; residual Cl may adsorb on some basic sites of Ca-P-O. The Cl/Pt ratio decreases slightly to 1.26 after H_2_ treatment, and remains at 1.22 after undergoing CO oxidation. The decrease in the Cl content after H_2_ treatment may account for the higher activity of Pt/Ca-P-O pretreated in 4% H_2_ (Figure S1).

### 2.7. Discussion

Much effort has been made in the literature to develop new catalysts with various compositions and tailored structures/morphologies, study the effect of preparation parameters, characterize the catalysts, and explore the application of the catalysts in various reactions. Most supported metal catalysts are composed of metals and solid supports. The solid supports are usually oxides (e.g., SiO_2_, Al_2_O_3_, TiO_2_, ZrO_2_, Fe_2_O_3_, CeO_2_) and carbons, but metal salts have been much less frequently used as supports. In the literature, some metal carbonates, phosphates, and sulfates have been used as supports to load metals [2]. From the examples on the catalytic applications of metal phosphate-based catalysts cited in the Introduction section [4,5,6,7,8,9,10,11,12,13,14,15,16,17,18,19,20,21,22,23,24,25,26,27,28,29,30,31,32,33,34,35,36,37,38,39,40,41,42,43,44,45,46,47,48,49,50,51,52,53,54,55,56,57,58,59,60,61,62,63,64,65,66,67,68,69,70,71,72,81,82,83], one can see that research in this direction is interesting, because different metal phosphates have different acid-base properties that may have an effect on theircatalytic performance.

To put our current work in perspective, it should be mentioned that Dai and co-workers [33,34] have previously developed a series of metal phosphate-supported Au catalysts for CO oxidation. The key point of that work is that some Au/M-P-O catalysts can also be active for CO oxidation, although most Au catalysts reported in the literature are composed of Au and oxides or carbon. Here we again developed a series of metal phosphate-supported Pt catalysts for CO oxidation. Although Pt catalysts are generally less active than Au catalysts (for instance, Au/Ca-P-O calcined at 500 °C reached complete CO conversion at 40 °C whereas Pt/Ca-P-O reached 100% CO conversion at 140 °C), the objective of our current work is not just to focus on catalytic activity, but to develop a series of phosphate-supported Pt catalysts that may be useful for CO oxidation and other reactions that may take advantage of the different acid-base properties of the metal phosphate supports.

Our current research has shown that Pt/M-P-O catalysts calcined at 300 °C and reduced in 4% H_2_ at 300 °C are active for CO oxidation (Figure 1). The T_50_ values of Pt/M-P-O (M = Mg, Al, Ca, Fe, Co, Zn, La) calcined at 300 °C and then pretreated in 4% H_2_ at 300 °C are 123, 128, 92, 123, 90, 130, and 123 °C, respectively. The difference of catalytic activities becomes more obvious when these catalysts were calcined at 500 °C. The T_50_ values of Pt/M-P-O (M = Mg, Al, Ca, Fe, Co, Zn, La) calcined at 500 °C and then pretreated in 4% H_2_ at 300 °C are 140, 178, 76, 120, 107, 202, and 123 °C, respectively. The activity follows the sequence of Pt/Ca-P-O > Pt/Co-P-O > Pt/Fe-P-O > Pt/La-P-O > Pt/Mg-P-O > Pt/Al-P-O > Pt/Zn-P-O. As shown in Figure 2, the calcination at 500 °C can lead to the sintering of Pt nanoparticles on some supports (*i.e.*, Mg-P-O, Fe-P-O, Co-P-O, and Zn-P-O). This can explain the decrease in activity for some catalysts calcined at 500 °C. In particular, Pt/Zn-P-O undergoes significant loss of activity upon calcination at 500 °C, probably due to the sintering of Pt nanoparticles.

Because the difference in catalytic activity is more obvious for the catalysts calcined at 500 °C, most of the characterization was focused on the catalysts calcined at 500 °C. XRD data show the presence of sharp Pt peaks for Pt/Mg-P-O, Pt/Fe-P-O, Pt/Co-P-O, and Pt/Zn-P-O calcined at 500 °C. TEM characterization indeed shows the presence of big Pt particles on the former three catalysts. The high-resolution TEM images of Pt/Zn-P-O cannot be obtained due to the transformation of the sample under high-energy electron beam. However, TEM characterization also shows the presence of a significant portion of small Pt particles on the former three catalysts. The TEM data are consistent with the XRD data.

XRD data show that there are no Pt peaks for Pt/Al-P-O, Pt/Ca-P-O, and Pt/La-P-O calcined at 500 °C, indicating the high dispersion of Pt nanoparticles on these supports. Our TEM data provide evidence for this conclusion. These highly dispersed Pt nanoparticles should be responsible for the catalytic activities. Note that the Pt loading on Pt/Ca-P-O (0.85%) is even lower than that on Pt/Al-P-O (1.35%), and the Pt particle sizes on both catalysts are similar, but Pt/Ca-P-O calcined at 500 °C is much more active than Pt/Al-P-O. Therefore, support effect plays an important role in influencing the catalytic activity.

The roles of solid supports are to disperse the active component (e.g., metal nanoparticles) and bestow surface areas, thermal stability, and mechanical strength to the prepared catalysts [1]. Some supports also have acid/base and/or redox properties, and therefore can facilitate certain catalytic reactions. One classical example is the isomerization of an *n*-paraffin to an isoparaffin over Pt/SiO_2_-Al_2_O_3_ [93]. While Pt is effective for the dehydrogenation and hydrogenation steps, the acidic support (SiO_2_-Al_2_O_3_) is responsible for the isomerization step.

For oxidation reactions such as CO oxidation, the nature of the oxide support is important. Some non-reducible oxides (e.g., SiO_2_, Al_2_O_3_) are classified into “inert” supports because they cannot supply oxygen for CO oxidation, whereas some reducible oxides (e.g., TiO_2_, Fe_2_O_3_, CeO_2_) are classified into “active” supports because they can supply lattice oxygen to react with CO, leaving defects that are then replenished by a separate reaction with oxygen from the gas phase [94,95]. In our case, H_2_-TPR data indicate that Mg-P-O, Al-P-O, Ca-P-O, Zn-P-O, and La-P-O supports are non-reducible, whereas Fe-P-O and Co-P-O supports are reducible (Figure S6). Therefore, although a reducible support may help the catalyst achieve high activity, this is not a prerequisite; the most active catalyst Pt/Ca-P-O has a non-reducible support.

Note that Ca-P-O (hydroxypatite) has rich basic sites and hydroxyls. Indeed, our CO_2_-TPD data indicate the presence of plenty of basic sites on Pt/Ca-P-O (Figure 6), and our XPS data indicate the presence of significantly more hydroxyls on Pt/Ca-P-O than on other catalysts (Table 1, Figure 8 and Figure 10). In the literature, both basic sites [96,97] and surface hydroxyls [40] were reported to facilitate catalytic CO oxidation. For instance, Lee *et al.* [96] found the addition of K_2_O or Na_2_O into Pt/Al_2_O_3_ can increase the number of basic sites thus increasing the activity in CO oxidation. Liu *et al*. [97] reported that the presence of basic sites on supports of Pd catalysts can facilitate the charge transfer in the catalyst and enhance the adsorption of CO, thus increasing the activity in CO oxidation. Chen *et al*. [89] demonstrated the presence of hydroxyls on Pt/Fe(OH)_x_ by XPS, and proposed, based on density function theory (DFT) calculations, that surface hydroxyls can combine with CO to form –COOH intermediate which then decomposes to CO_2_ and interfacial vacancies. The vacancies are prone to adsorbing O_2_ to form –OOH which can react with CO to form CO_2_, recovering surface hydroxyls [89]. The beneficial effect of surface hydroxyls in facilitating CO oxidation over Au/SiO_2_ was also reported by Wu *et al*. [98]. Although here we do not have clear and direct evidence to show how surface hydroxyls are involved in the reaction mechanism and how surface basic sites may promote the reaction in our case, the correlation between surface hydroxyls/basicity and catalytic activity is clear. We hope that future studies can address these points employing more advanced methods, such as DFT calculation, kinetics studies, and isotope-labeling experiments.

It should be noted that Pt/La-P-O has a larger surface area, more basic sites, and higher Pt content than Pt/Ca-P-O (see Table 1), but its activity is significantly lower. We believe that this is due to two factors. First, as shown in Figure 6, Pt/La-P-O has a desorption peak centered at 257 °C whereas Pt/Ca-P-O has a main desorption peak centered at 204 °C. The amounts of basic sites are 147.9 and 76.8 μmol/g, respectively. That means that Pt/La-P-O not only has more basic sites than Pt/Ca-P-O, its basic strength is stronger than that of Pt/Ca-P-O. Too strong and too many basic sites of Pt/La-P-O may make the desorption of CO_2_ (produced by CO oxidation) difficult. As a rule of thumb in heterogeneous catalysis, too strong adsorption of the reactant/intermediate/product usually lowers the activity in catalysis [1]. Second, the hydroxyl level of Pt/La-P-O (8.8% among all surface oxygen species) is significantly lower than that of Pt/Ca-P-O (37.9%, see Table 1).

Although here we chose CO oxidation as a probe reaction, we believe than the newly developed Pt/M-P-O catalysts may be useful in other reactions. From the examples cited in the Introduction section, it can be predicted that the most probable field the Pt/M-P-O catalysts can be useful is organic catalysis because Pt can catalyze hydrogenation/dehydrogenation reactions, whereas different metal phosphates have different acid-base properties and hydroxyapatite also has rich hydroxyls. The combination of both functionalities furnished by both Pt and M-P-O supports can make the catalysts be useful in organic catalysis and other reactions. This should be studied in more details in the future.

## 3. Experimental Section

### 3.1. Synthesis of Catalysts

H_2_PtCl_6_·6H_2_O (Sinopharm Chemical Reagent, AR, Shanghai, China), Mg_3_(PO_4_)_2_·xH_2_O (Aldrich, >98%, Shanghai, China), AlPO_4_ (Aladdin, CP, Shanghai, China), Ca_5_(OH)(PO_4_)_3_ (J&K Chemical, 96%, Shanghai, China), FePO_4_·xH_2_O (Alfa Aesar, CP, Shanghai, China), CoPO_4_ (Alfa Aesar, 98%), Zn_3_(PO_4_)_2_ (J&K Chemical, CP), and LaPO_4_·xH_2_O (Alfa Aesar, 99.99%) were used as received. The Pt/M-P-O catalysts were prepared by a conventional incipient wetness impregnation method. In a typical synthesis, 0.212 g H_2_PtCl_6_·6H_2_O was dissolved in 40-50 mL deionized water in a 250 mL beaker; 3.92 g M-P-O was then weighed and immersed into the aqueous solution at room temperature. The mixture was stirred gently using a glass rod and allowed to keep static overnight, and then dried in an oven at 80 °C for 12 h. The obtained solid was grid into powders and divided into two parts which were then calcined in a muffle oven under static air at 300 or 500 °C, respectively, for 2 h.

### 3.2. Characterization

XRD experiments involving spent Pt/M-P-O catalysts (calcined at 300 or 500 °C, pretreated in 4% H_2_ at 300 °C, and then tested in CO oxidation) and M-P-O supports calcined at 500 °C (for reference) were conducted on a PW3040/60X'Pert PRO X-ray diffractometer (PANalytical, Almelo, Netherlands) with Cu Kα radiation at 40 kV and 40 mA. The scanning was conducted at 2θ = 20–90°, and the scanning rate was 6°/min.

The specific surface areas (S_BET_) of Pt/M-P-O calcined at 500 °C were determined from nitrogen adsorption-desorption isotherms measured at −196 °C using a Micromeritics ASAP 2020 M+C surface area and porosity analyzer (Micromeritics, Norcross, GA, USA). Before the measurement, a sample (0.1–0.2 g) was degassed at 200 °C for 6 h, the weight of the sample was measured again, and the sample was then subjected to N_2_ adsorption-desorption.

ICP analyses of Pt/M-P-O catalysts calcined at 500 °C were conducted using a Model-P4010 instrument (Hitachi, Tokyo, Japan) after dissolution of the samples in aqua regia and appropriate dilution.

TEM experiments involving spent Pt/M-P-O catalysts (calcined at 300 or 500 °C, pretreated in 4% H_2_ at 300 °C, and then tested in CO oxidation) were conducted on a JOEL JEM2100F field-emission transmission electron microscope (JOEL Ltd., Tokyo, Japan) with acceleration voltage of 200 kV. Prior to recording TEM images, a small portion of sample was dispersed in ethanol, and a few drops were dropped onto a Cu grid-supported carbon film and dried under an infrared lamp.

CO_2_-TPD experiments involving Pt/M-P-O catalysts (calcined at 500 °C, pretreated in 4% H_2_ at 300 °C) were performed in a FINESORB-3010 temperature-programmed chemsorber (FINETEC, Hangzhou, China). A catalyst (0.25 g, 40–60 mesh) was loaded into a U-shaped glass tube, 4% H_2_ (balance He, total flow rate 50 mL/min) was then used to sweep the catalyst, and the temperature was ramped from room temperature to 300 °C (at a rate of 10 °C/min) in the presence of flowing 4% H_2_, and kept at 300 °C for 2 h. Then the catalyst was cooled to 50 °C, and exposed to flowing CO_2_ (40 mL/min) at 50 °C for 1 h. The CO_2_ gas was then switched to He (40 mL/min) and swept by the He flow for 3 h to remove physically adsorbed CO_2_. The temperature was then ramped to 600 °C at a rate of 10 °C/min to allow for the desorption of adsorbed CO_2_ under flowing He.

XPS experiments involving spent Pt/M-P-O catalysts (calcined at 500 °C, pretreated in 4% H_2_ at 300 °C, and then tested in CO oxidation) and additionally, Pt/Ca-P-O calcined at 500 °C as well as Pt/Ca-P-O calcined at 500 °C and pretreated in 4% H_2_, were collected on a Perkin-Elmer PHI 5000C ESCA instrument (Perkin Elmer, Waltham, MA, USA), with MgKα radiation source (voltage 14 kV, current 20 mA). The binding energies were corrected by using the binding energy of C1s as a reference, and the XPS data were processed using XPS PEAK41 software.

H_2_-TPD experiments involving M-P-O supports calcined at 500 °C were conducted in a FINESORB-3010 temperature-programmed chemsorber (FINETEC, Hangzhou, China). A sample (0.12 g, 40–60 mesh) was loaded into a U-shaped glass tube, and the sample was then heated from room temperature to 50 °C at a rate of 10 °C/min under the protection of flowing He. The gas was then switched to 10% H_2_ (balance Ar, total flow rate 30 mL/min), and the sample was swept by 10% H_2_ for 3 h. Finally, the temperature was ramped from 50 to 800 °C, and 10% H_2_ was still flowing at a rate of 30 mL/min.

### 3.3. Catalytic Testing

A catalyst (0.25 g) was loaded into a U-shaped glass tube, sealed by quartz wools on both sides of the catalyst zone, and the U-shaped glass tube was then put in a fixed bed reactor (FINESORB-3010 temperature-programmed chemsorber, FINETEC, Hangzhou, China), and 4% H_2_ (balance He) was flowed through the catalyst at a rate of 50 mL/min. The catalyst was heated to 300 °C at a rate of 10 °C/min, and kept at 300 °C for 2 h, while the 4% H_2_ was still flowing. After the catalyst was cooled down to room temperature, the gas stream was switched to 1% CO (in air, flow rate: 50 mL/min). The catalyst was maintained at room temperature for 1 h, and then heated to 300 °C at a rate of 0.5 °C/min, while the 1% CO was still flowing at a rate of 50 mL/min. The space velocity was 12,000 cm^3^/(h·g_cat_). The exiting stream was continuously analyzed by GC (Agilent 7890A). The CO conversion was calculated as [CO_2_]_out_/([CO]_out_ + [CO_2_]_out_).

## 4. Conclusions

A series of metal phosphate-supported Pt catalysts (Pt/M-P-O, M = Mg, Al, Ca, Fe, Co, Zn, La) were developed and tested for CO oxidation. The catalysts calcined at 300 °C and pretreated in 4% H_2_ at 300 °C can achieve complete CO oxidation at 100–140 °C, whereas the catalyst calcined at 500 °C and pretreated in 4% H_2_ at 300 °C can achieve complete CO oxidation at 80-210 °C. The activity in the latter set of experiments follows the sequence of Pt/Ca-P-O > Pt/Co-P-O > Pt/Fe-P-O > Pt/La-P-O > Pt/Mg-P-O > Pt/Al-P-O > Pt/Zn-P-O. The better activity of Pt/Ca-P-O is not only related to the small Pt nanoparticles (2–3 nm) on Ca-P-O, but also linked to the basicity and rich hydroxyls on the support, as proved by CO_2_-TPD and XPS characterization. In addition, XPS data demonstrate that the pretreatment of Pt/Ca-P-O in 4% H_2_ can reduce Pt^4+^, remove a small portion of residual Cl, and enrich the hydroxyls. These effects are beneficial for enhancing the activity in CO oxidation. Although here we chose CO oxidation as a probe reaction, we believe than the newly developed Pt/M-P-O catalysts may find applications in other reactions, considering the functionality furnished by both Pt and M-P-O supports.

## Supplementary Materials

Supplementary materials can be accessed at: http://www.mdpi.com/1996-1944/7/12/8105/s1.
