# Supplementary material for: Metal Phosphate-Supported Pt Catalysts for CO Oxidation
Source: Materials (Basel). 2014 Dec 17;7(12):8105–30. doi: 10.3390/ma7128105 (PMC5456429; doi:10.3390/ma7128105)

Supporting Information

Figure S1. Effect of pretreatment on the catalytic performance of Pt/Mg-P-O (**A**); Pt/Al-P-O (**B**); and Pt/Ca-P-O (**C**). These catalysts, previously calcined at 500 °C, were either pretreated in 4% H_2 at 300 °C prior to catalytic testing (●), or not pretreated in 4% H_2 at all (■).

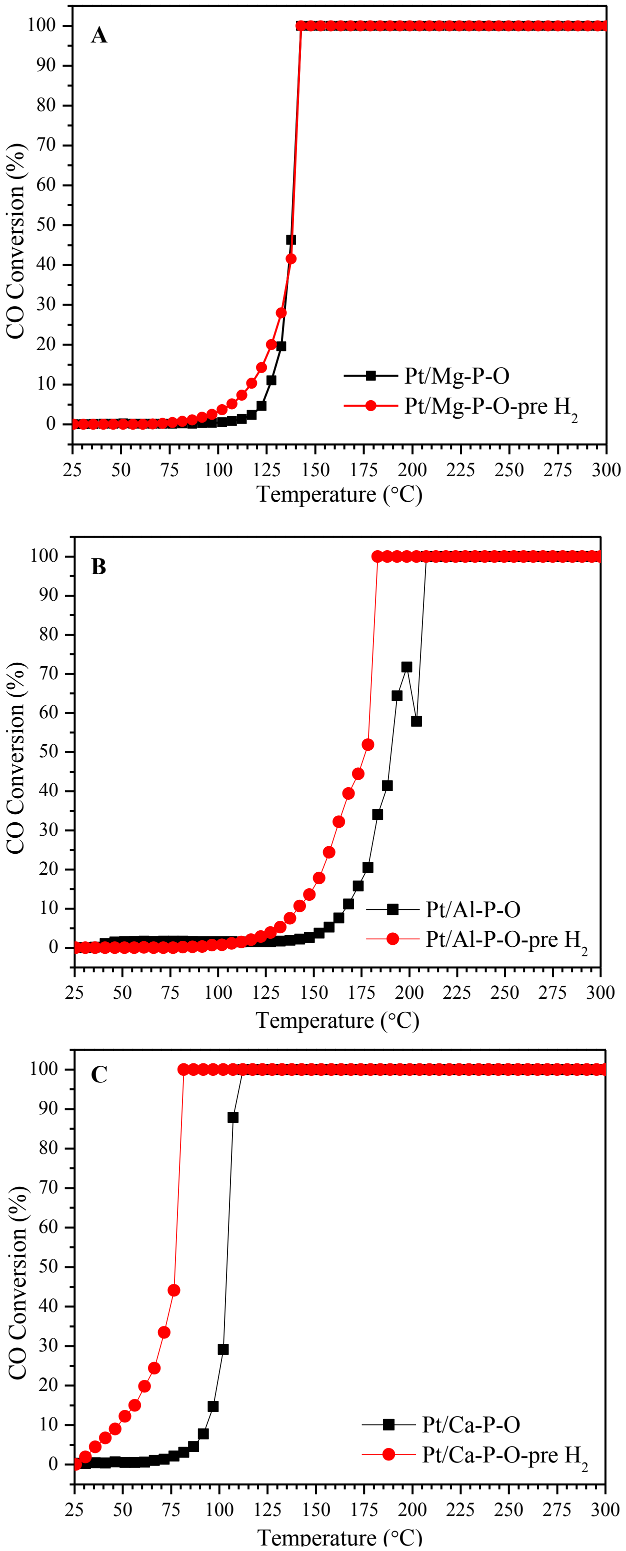

Figure S2. TEM image of spent Pt/Fe-P-O (calcined at 500 °C, pretreated in 4% H_2 at 300 °C, and tested in CO oxidation). Both images (**a**,**b**) correspond to the same catalyst.

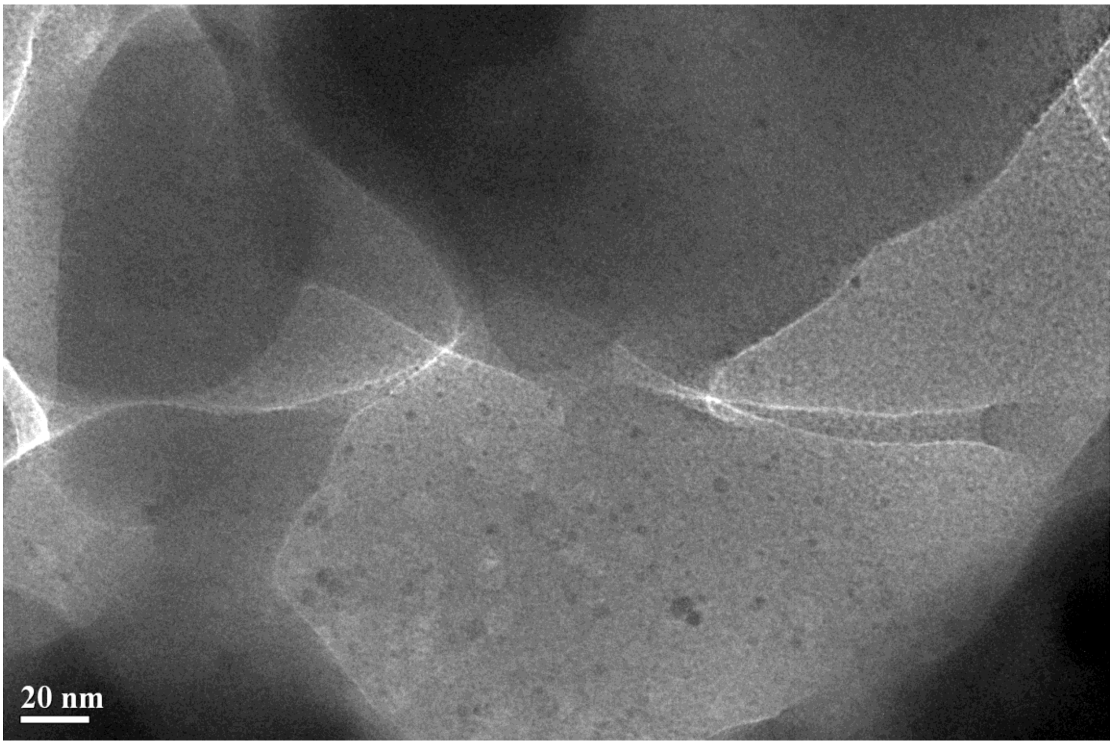

(**a**)

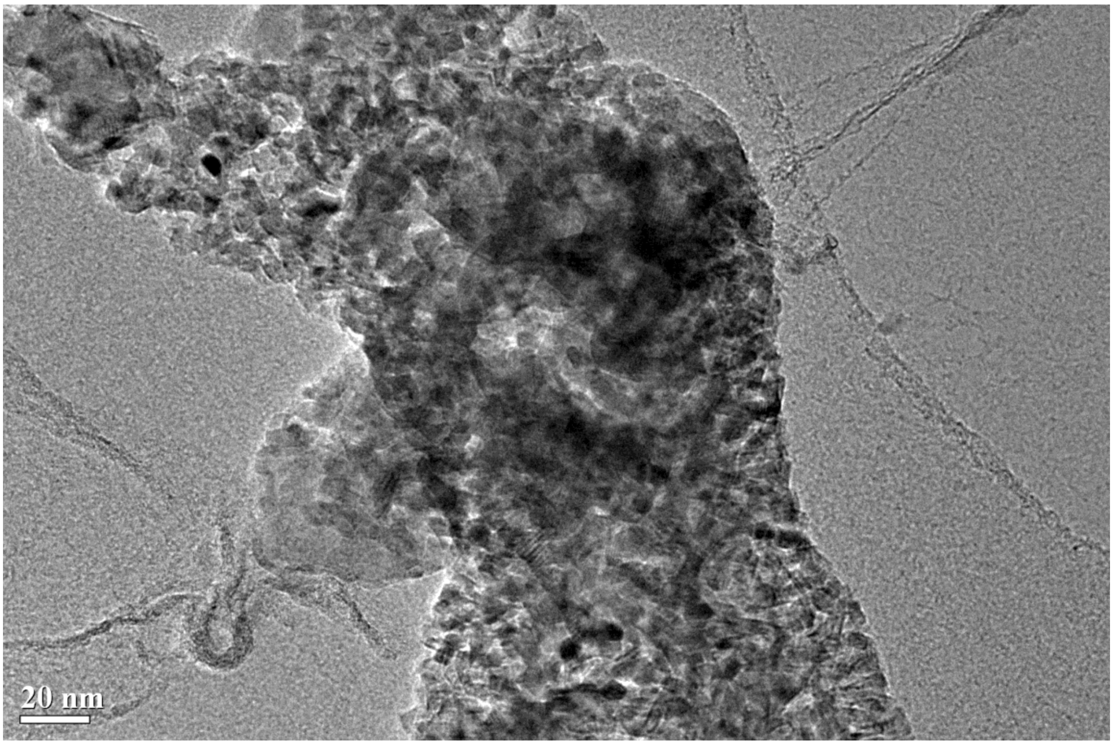

(**b**)

Figure S3. TEM image of spent Pt/Co-P-O (calcined at 500 °C, pretreated in 4% H_2 at 300 °C, and tested in CO oxidation). Both images (**a**,**b**) correspond to the same catalyst.

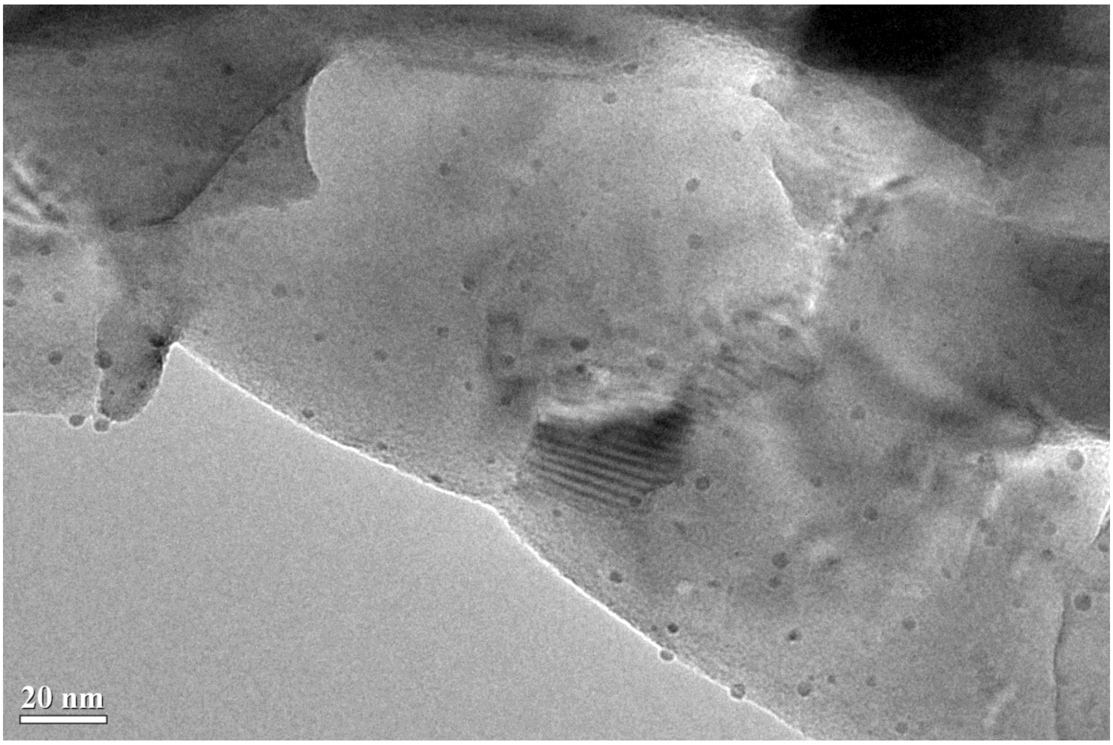

(**a**)

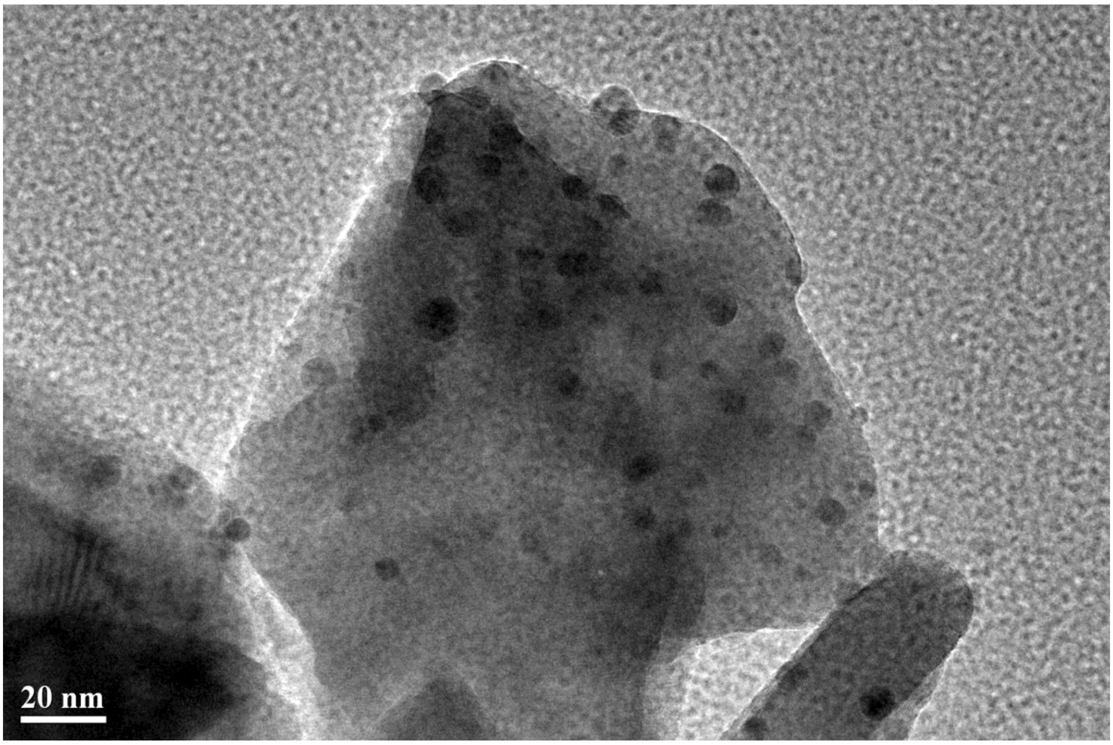

(**b**)

Figure S4. TEM image of spent Pt/Zn-P-O (calcined at 500 °C, pretreated in 4% H_2 at 300 °C, and tested in CO oxidation). Both images (**a**,**b**) correspond to the same catalyst.

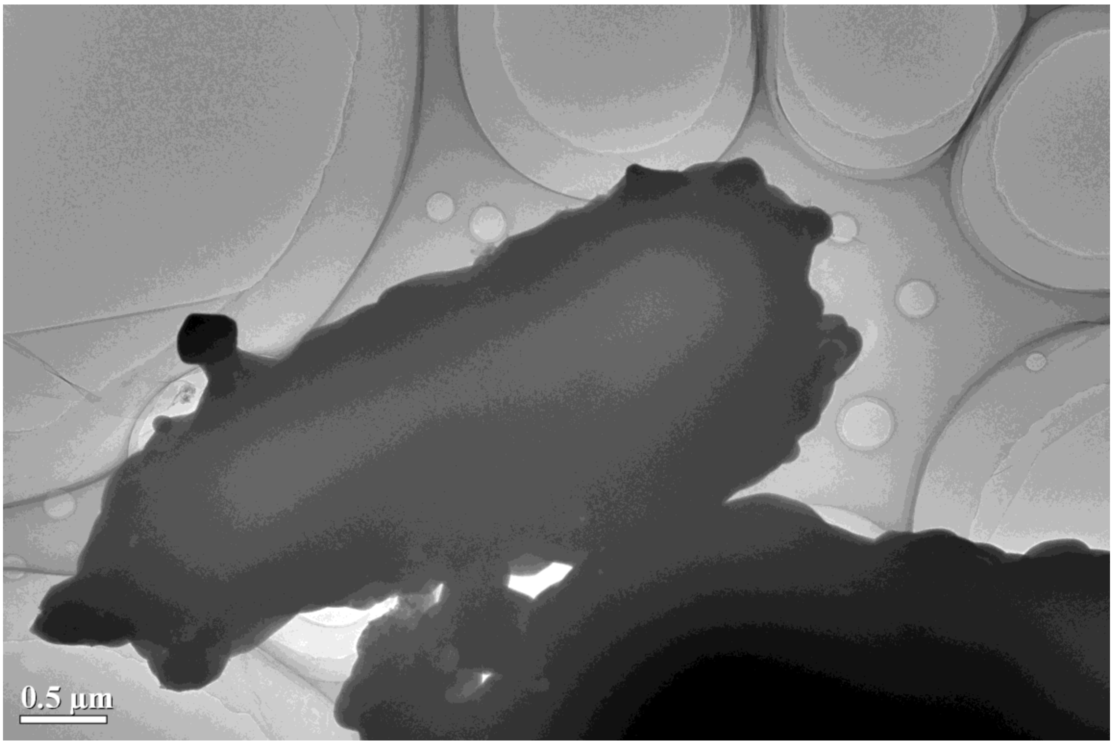

(**a**)

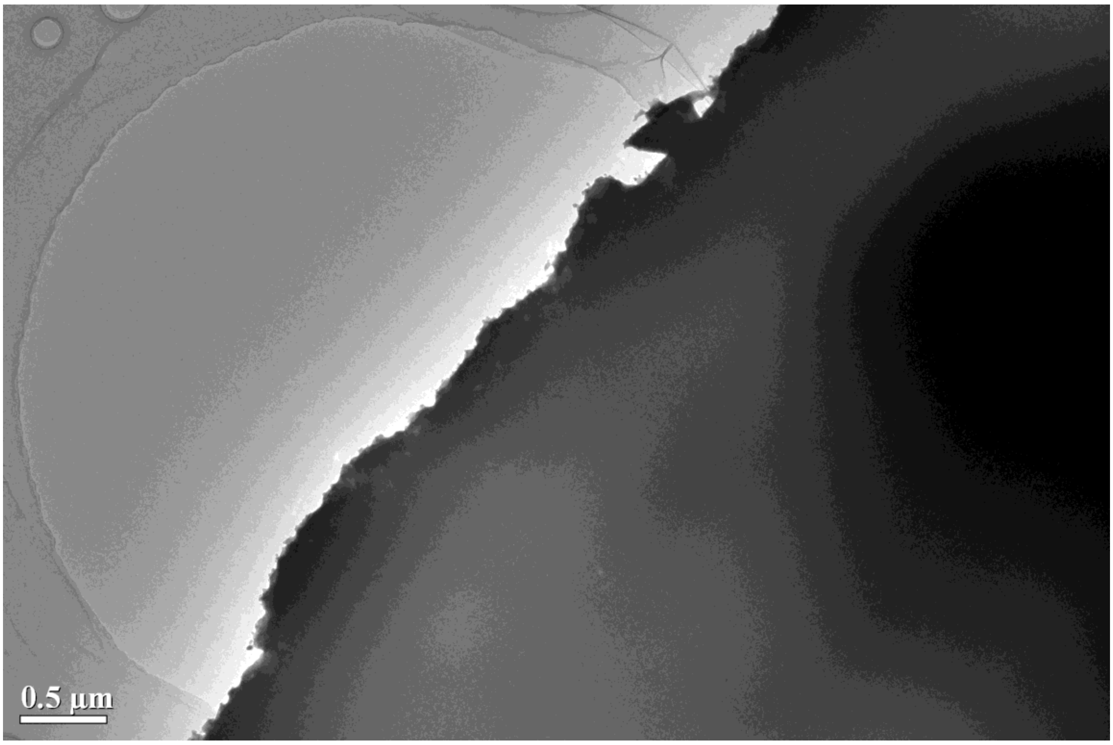

(**b**)

Figure S5. TEM image of spent Pt/La-P-O (calcined at 500 °C, pretreated in 4% H_2 at 300 °C, and tested in CO oxidation). Both images (**a**,**b**) correspond to the same catalyst.

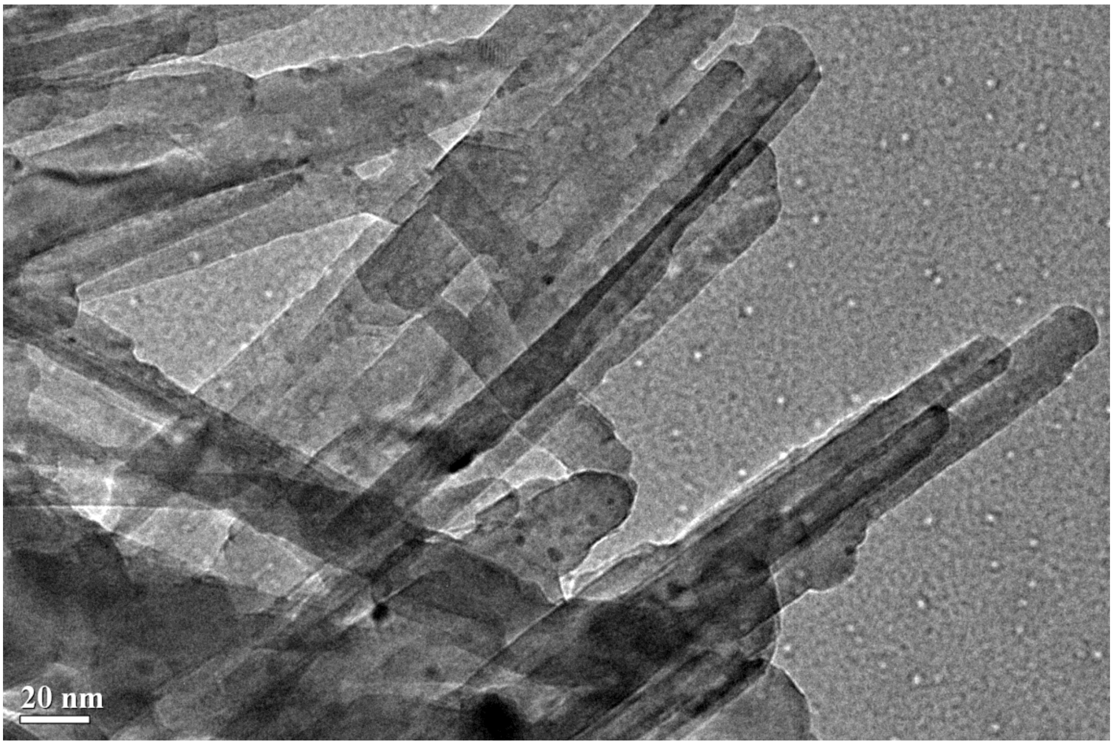

(**a**)

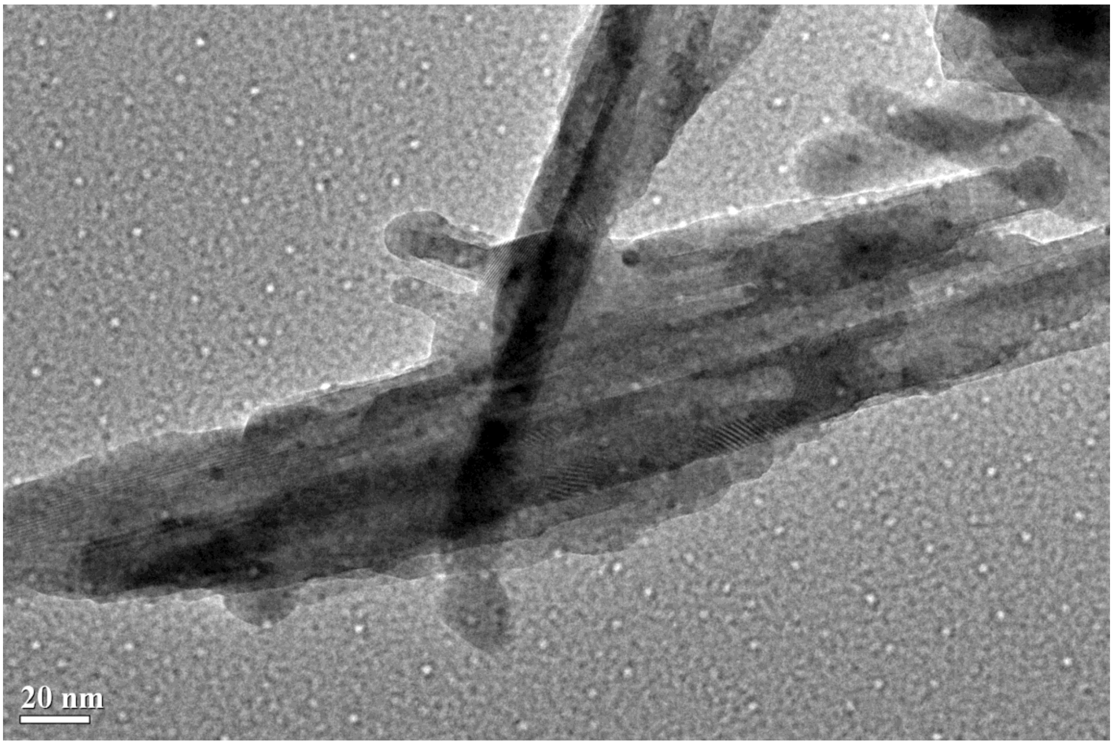

(**b**)

Figure S6. H_2-TPR data of M-P-O supports (calcined at 500 °C).

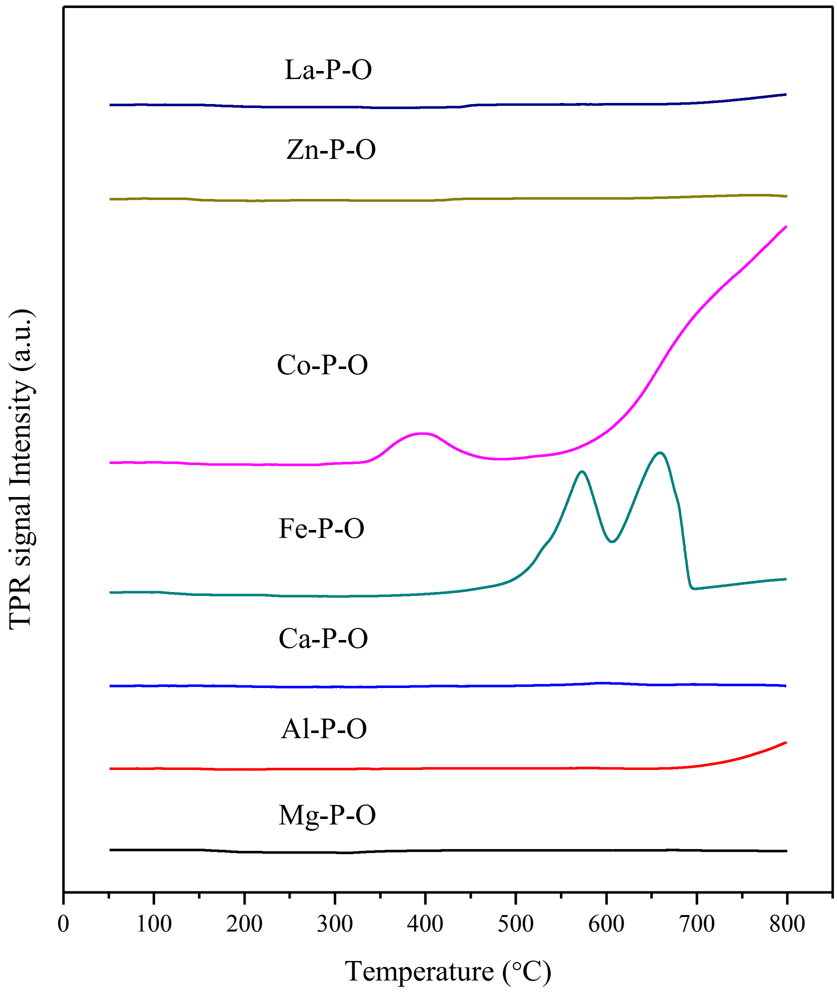

Supplement: Supplementary file 1 [file materials-07-08105-s001.pdf]
